# Book Reviews

**Published:** 1906-04

**Authors:** 


### BOOK REVIEWS.

The Psychic Treatment of Nervous Disorders. By Dr. Paul DuBois, Professor of Neuropathology, University of Berne. Translated by Smith Ely Jelliffe, M. D., Ph. D., and William A. White, M. D. 8vo., cloth; 471 pages. Price, $3.00 net. Funk & Wagnalls Company: New York and London. 1905.

This English translation by Jelliffe and White of DuBois' well-known book deserves to receive in this country the same degree of popularity it met with in France. It is a book that many have been waiting for. It presents in detail the methods of one of the most skillful advocates of what may be termed mental therapeutics, meaning by that, the understanding and the use of methods aimed to correct and educate and to persuade without the use of anything mystifying, nor of what is usually termed "suggestive." The book consists of a psychological introduction, written in a simple and easily understood manner, upon which the author is supposed to base his psychotherapy. This introduction is interesting, not on this account, but because it is so pleasingly written, and because it contains so much evidence that the author regards his psychology as a thing lying close to the every-day working plan of a neurologist. This attitude is a safe and hopeful one, at any rate, even if it does not tend to supply new psychological data. The description of cases is clear, and we really are able to put ourselves in the place of the patient as well as the physician, very often. This book is recommended to all neurologists, as well as to those who have any sort of interest in the more advanced methods in use in neurological therapeutics. The translation is very well done, and adds much to the delight which one feels in reading the book.

ANESTHETIS AND ANESTHETICS. By J. M. Patten, M. D., Professor of Physical Diagnosis and General Anesthesia in the College of Dentistry of the University of Illinois; Professor of Diseases of the Chest in the Chicago Polyclinic; Associate Professor of Medicine in the Medical Department of the University of Illinois. The volume consists of over 200 pages, octavo, on heavy, superior cream book-paper, is very handsomely bound in green or tan half-calf, with gilt top, and sold, delivered, for $2.50 net. Publishers, The Cleveland Press, Chicago.

There has been nothing of this kind brought out in America, and for long such a book has been wanted by the active man who had neither the time nor facilities for searching through current periodical literature for the advancements that have been made, or for the best conclusions and the best medico-legal positions touching anaesthetic drugs and procedures in their use.

Perhaps no dental subject is more important than this one, for while anaesthesia constitutes one of the most radical blessings of modern discovery at the same time there are definite limitations of use, positive dangers, and many very certain and well-tried directions in the matter of means and procedure that must be well understood in order to render the employment of this class of substances (anaesthetics) both safe and efficacious.

All such information, and much of a correlated nature, is set forth in this volume by one skilled in logical analysis and at the same time one who has had a wide experience in the administration of the agents used.

General and local anaesthesia and anaesthetics are both thoroughly covered.

